# Genomic analysis of the rare British Lop pig and identification of distinctive genomic markers

**DOI:** 10.1371/journal.pone.0271053

**Published:** 2022-08-12

**Authors:** Georgios Banos, Andrea Talenti, Dimitrios Chatziplis, Enrique Sánchez-Molano

**Affiliations:** 1 Scotland’s Rural College (SRUC), Department of Animal and Veterinary Sciences, The Roslin Institute Building, Edinburgh, United Kingdom; 2 The Roslin Institute and R(D)SVS, University of Edinburgh, Edinburgh, United Kingdom; 3 Laboratory of Agrobiotechnology and Inspection of Agricultural Products, Department of Agriculture, International Hellenic University, Sindos, Greece; University of Iceland, ICELAND

## Abstract

Concentration of production on a few commercial pig breeds has led to the marginalization of many native, numerically small breeds, increasing their risk of endangerment. In the UK, one such rare breed is the British Lop, a lop-eared breed, of similar origin to the Welsh breed. The objective of the present study was to address the genomic status of the British Lop and its relationship with other breeds and identify a small set of genomic markers that uniquely characterize and distinguish British Lop animals. Results have shown that the British Lop is a relatively distinct population with reduced genomic diversity and effective size consistent with its status as a rare breed. Furthermore, we demonstrated the genetic closeness of the British Lop to phenotypically similar breeds such as Landrace and Welsh as well Large White, Middle White and Pietrain. Finally, a set of 75 Single Nucleotide Polymorphisms distributed across multiple chromosomes were identified and validated as markers that can consistently distinguish British Lops from other closely related breeds. Results may inform breeding and management strategies aiming to enhance diversity as well as the development of a breed purity test.

## Introduction

Globalization in the latest decades has led to an increasing demand of livestock products, with the pig industry accounting for between 35% and 40% of the worldwide meat production [1]. Increasing consumer demand has led to intense changes in industry, with mainly a few international commercial breeds being extensively used and improved through merging and consolidation of the most successful breeding lines [2]. In the UK, commercial pigs are mainly crosses between Landrace and Large White, with contributions from other breeds such as Hampshire, Pietrain, Danish Duroc and British Saddleback [3].

At the same time, several traditional autochthonous breeds have been marginalized, leading to endangerment according to the Rare Breeds Survival Trust (RBST); examples of such breeds include the British Lop, Tamworth and Berkshire [4]. However, the maintenance of these rare, numerically small breeds is of paramount importance. From a genetic point of view, rare breeds and populations can act as resources of genetic variability and might be adapted to particular environmental conditions. With current climate change trends leading to increased average temperatures and weather variation, genetic diversity and the ability of animals to successfully respond to external challenges become especially important. As such, rare breeds could be utilized in the main commercial lines to prevent an excessive decay in genetic variation that could endanger animal adaptability. Furthermore, rare breeds are also important from a cultural point of view, as they have characteristics that are valued due to specific regional and historical connections and may also provide quality products for niche markets.

The use of genomics may be particularly helpful to farmers and breed societies. For example, the use of genomic data can complement the information provided from existing pedigrees, to increase the accuracy of inbreeding and effective population size estimates and underpin relevant genomic breeding schemes. Furthermore, genomics tools can also be used to confirm if individual animals belong to a certain breed. In a previous study [5], a genomic tool was developed for product regulation in the UK pig market, using the commercial Illumina PorcineSNP60 beadchip, and including several traditional pig breeds such as Berkshire, British Saddleback and Welsh. In a subsequent study [6], the same commercial SNP array was used to identify selection signatures in these breeds. The Illumina commercial SNP array was also used to estimate genomic relationships and within-breed variation in some rare pig breeds, including British Saddleback and Tamworth [7]. Further studies have also addressed the detection of selection signatures and the evaluation of genomic diversity in autochthonous European pig breeds using either commercial genome-wide SNP arrays or whole-genome sequencing [8–12].

The British Lop pig is a numerically small native breed [13] classified as a ‘breed at risk’ by the UK Department for Environment, Food & Rural Affairs [14]. The breed emerged from crosses between different local lop-eared breeds at the end of the XIX and beginning of the XX centuries. While other breeds have been influenced by Asian breeds imported into the UK in the early XIX century [15], the original breeds crossed to create the British Lop are thought to have remained relatively unaffected [16]. In 1956, the decision to concentrate improvement mainly on Landrace, Welsh and Large White following the Howitt report [17] led to a lack of support for the breed, resulting in a reduced population size and geographic isolation to the Southwest of England. Since 1960, the actual population size has slowly stabilized, although still remains amongst the numerically smallest of the native UK breeds, with about 161 sows registered in 2019 [13]. Previous studies have shown low levels of genetic exchange between the British Lop and commercial UK breeds, indicating a great potential and need to maintain the unique genetic characteristics of the British Lop and improve productivity of current UK commercial populations with crossbreeding [13]. There is a need to safeguard the genetic integrity of the breed. No genomic studies on this breed have been conducted before.

The present study aims to genomically explore the British Lop, one of the rarest UK native breeds and provide insights that may underpin future breeding schemes. Three main specific objectives are addressed within this aim: i) perform a genomic characterization of the British Lop breeding population; ii) investigate the genomic relationship of the British Lop with commercial populations and other UK pig breeds and iii) identify a set of genomic markers that distinguish the British Lop pigs from other breeds.

## Material and methods

### Animal data and genotypes

Hair samples were obtained from 190 British Lop pigs from 40 farms that are members of the British Lop Pig Society. These animals represent a significant cross-section of the current breeding population. All available animal pedigree records were also obtained, consisting of 2,901 records including historic data.

Hair samples were genotyped using the Illumina PorcineSNP60v2 BeadChip, comprising 61,565 Single Nucleotide Polymorphisms (SNPs) spread throughout the entire genome.

Additional genotypes of 348 pigs representing 13 other UK pig breeds and commercial populations were obtained from the public domain (https://doi.org/10.5061/dryad.c2124) [6], including Berkshire (30 pigs), British Saddleback (30), Duroc (26), Gloucestershire Old Spots (24), Hampshire (30), Landrace (27), Large Black (30), Large White (31), Middle White (30), Pietrain (26), Tamworth (30) and Welsh (34). Twenty nine (29) wild boar genotypes were also added.

During the merging process of the British Lop genotypes with those of the above populations, map positions were updated consistently with the *Sus scrofa* 11.1 genome assembly [18] but alleles were subsequently reported according to the Forward strand in the 10.2 assembly.

An initial exploration of the British Lop data was performed to identify low quality genotypes and duplication errors. SNP quality control using PLINK [19] removed SNPs on sex chromosomes, non-mapped SNPs, SNPs with call rates lower than 90% and SNPs with a minor allele frequency lower than 0.02 [10]. Further edits also removed samples with call rates lower than 90% as well as potentially duplicate samples with pairwise Identity-by-State (IBS) greater than 98%. As a result, eight low quality genotypes and four duplicated samples were removed from the ensuing analyses.

In addition, the genomic relationship matrix among animals calculated with the remaining SNPs was used in a principal component analysis using GEMMA [20]. A visual inspection of the latter revealed two British Lop samples that were not clustering with the rest of the population. These animals were also removed from most data analyses but used in the validation process described later.

For consistency, the edits described above for the British Lop were applied to the genotype data pertaining to the other breeds too.

### Genomic characterization of the British Lop

The first aim of the present study was to perform a genomic characterization of the British Lop population and estimate measurements of genetic diversity including genomic and pedigree inbreeding levels. The following analyses were conducted:

#### Estimates of genomic diversity and runs of homozygosity

Since the 0.02 threshold for the minor allele frequency could possibly bias the genomic diversity estimates, this edit did not apply here. Therefore, after excluding the two British Lop samples not clustering with the rest of the population, the dataset for these analyses comprised 176 British Lop samples genotyped with 44,315 SNPs.

Estimates of Tajima’s D and nucleotide diversity were obtained using VCFtools [21] over windows of 1 Megabase (Mb). This size was in concordance with the minimum length chosen for the analyses of runs of homozygosity (ROH), which were computed using PLINK [19]. Given the extent of linkage disequilibrium (LD) in the breed of study, due to genetic drift and/or selection, the chosen size is not expected to cause biases in the diversity estimates. The minimum number of SNPs constituting a ROH was estimated as 55, following the formula of Lencz et al. [22] adapted by Purfield et al. [23], with the average frequency of heterozygous animals for all SNPs in the dataset being 0.2895 and using a value of alpha of 0.05. Other input parameters were the maximum allowance for one heterozygous and two missing genotypes per window, a minimum ROH length and a maximum gap between consecutive SNPs of 1 Mb, a minimum density of 1 SNP per 500 Kilobases (Kb), a scanning window size of 55 SNPs [24], and a scanning window threshold of 0.05.

Subsequently, individual ROH-based inbreeding (F_ROH_) was estimated as F_ROH_ = L_ROH_ /L_AUTO_, where L_ROH_ is the total length of ROH on autosomes and L_AUTO_ is the total length of the autosomes covered by SNPs [25], with the latter being calculated as 2,255,725.81 Kb.

#### Estimates of genomic and pedigree-based inbreeding and effective population size

The minor allele frequency edit (0.02) described above was applied in this step to account for genotyping errors, leading to a dataset of 176 genotypes and 36,048 SNPs.

In-house software was used to estimate the genomic relationship matrix calculated with the SNP information, and individual inbreeding coefficients were calculated from its diagonal. Two methods were used for this purpose: i) the VanRaden’s method 2 [26], assuming allelic frequencies of 0.5 in the base population [27] and ii) the allelic similarity method proposed by Nejati-Javaremi et al. [28]. Individual pedigree-based inbreeding coefficients were estimated using the R-package “pedigree” [29].

The historic effective population size was estimated from SNP data using the SNeP software [30] with the mapping function of Sved & Feldman [31]. Estimates of the effective population size using pedigree data were obtained from complete generations using the ENDOG v4.8 software [32]. A complete generation was defined as the number of generations separating an individual from the farthest ancestor, where all other ancestors of the individual were known.

### Relatedness among breeds

The second aim of the present study was to assess the genomic relationship of the British Lop with other UK pig breeds and populations. This was achieved by i) inspecting the IBS genetic distances between the different breeds, ii) identifying potential historic migration events that might have taken place between breeds and iii) inferring the current degree of admixture per individual and the number of ancestral breeds.

In order to avoid bias due to the over-representation of the British Lop breed, 33 representative British Lop samples were kept for these analyses. These samples were selected using the Multi-Dimensional Scaling analysis implemented in the R-package “BITE” through the function “representative.sample” [33]. Default parameters were used (similarity threshold of 0.75, 3 dimensions, 95% individual call rate, 95% acceptable threshold value for IBS, 3,000 markers sampled at random and 0.01 false discovery rate for unacceptably high individual heterozygosity) together with a maximum of 1,000 iterations. To confirm the representativeness of the selected samples, a cluster analysis was performed and visually inspected.

Subsequently, the 33 representative British Lop samples were combined with genotypes from the other UK traditional and commercial breeds described above (between 24 to 34 samples per breed). Quality control for this combined dataset included the removal of SNPs with minor allele frequencies lower than 0.02. To avoid potential bias due to missing genotypes and strong LD among SNPs, LD pruning was performed using PLINK [19] assuming an r^2^ threshold of 0.3, window sizes of 50 SNPs and shifting steps of five SNPs. Furthermore, SNPs with at least one missing genotype were removed using VCFtools [21]. These steps resulted in a dataset containing 405 samples and 14,628 SNPs, which were used for the following three analyses:

#### Neighbor-joining IBS consensus tree

IBS genetic distances between samples were estimated in PLINK [19] for 100 bootstrap permutations and used as input for the software PHYLIP [34]. A neighbor-joining consensus tree with wild boar as the outgroup was built and plotted using the software GraPhlAn [35], with clades assigned to individual pigs and colored by breed.

#### Maximum-likelihood tree with migration edges

Allele frequencies for each breed were estimated in PLINK [19] and used as input to the Treemix software [36] to construct a maximum likelihood consensus tree including migration edges. The optimal number of migration edges was determined using 10 bootstrap replicates per node number and the Evanno method [37] implemented in the R-package “OptM” [38]. Two maximum likelihood consensus trees were then constructed assuming 4 and 12 migration edges with 100 bootstrap replicates, using permutation blocks of five SNPs (given the LD pruning performed during the quality control process) and considering wild boar as the outgroup root. The output was plotted using the function “treemix.bootstrap” in the R-package “BITE” [33].

#### Unsupervised admixture analysis

The degree of admixture per individual and the number of ancestral breeds were assessed with the maximum likelihood method implemented in the software ADMIXTURE [39]. To estimate the most likely number of ancestral populations (K), error estimates were calculated using a 5-fold cross-validation for different values of K ranging from 1 to 17. The label was given a posteriori by comparing the membership coefficient with the known breed. Results of the analyses were plotted using the function “membercoeff.circos” from the R-package “BITE” [33].

### Identification of a unique SNP set distinguishing the British Lop

The third aim of the present study was to identify a unique subset of SNPs that could be easily used to distinguish the British Lop from the other UK breeds included in this study.

Twenty representative samples were selected from each breed to constitute the training set, using the Multi-Dimensional Scaling approach described above. Sixteen additional independent validation sets were created, not containing any samples present in the training set. Each of these validation sets included British Lop samples randomly chosen plus 6–14 samples per each of the other breeds. Furthermore, the last two validation sets also included one of the two British Lop outliers identified in the initial PCA of genotypes.

When comparing two populations, a common procedure involves selecting SNPs that are likely to be fixed within one of them. However, the fixation index alone may not be the appropriate way to assess marker informativeness when the number of populations is greater than two [40]. As the present study included animals from multiple populations, marker alleles may have been fixed in more than one population due to genetic drift. Therefore, instead of fixed SNPs, we focused on segregating SNPs with high variability, for which different combined distributions of allelic frequencies may be observed in the different populations. Quality control on the training set removed samples and SNPs with call rates lower than 90% and SNPs with minor allele frequencies lower than 0.3. Furthermore, additional quality control included strong LD pruning (assuming an r^2^ threshold of 0.2, window sizes of 50 SNPs and shifting steps of five SNPs) and removal of all SNPs with at least one missing genotype. These edits led to a training dataset containing 120 genotypes and 3,417 variants.

In the first step to identify the best markers that distinguish British Lop animals from the others, low-informative markers were removed following pairwise F_st_ analyses between British Lop and every other breed in the training dataset using PLINK [19]. In each pairwise analysis, the top 5% of SNPs with the greatest F_st_ were selected, separating British Lop from at least one other breed. In the second step, these preselected SNPs were further filtered by keeping the smallest combination of SNPs that fully distinguished the British Lop from all other breeds. This was achieved with a Canonical Discriminant Analysis within each chromosome, using the R-package “MASS” [41]. In these analyses, and for canonical functions clearly separating the British Lop from all other breeds, only the SNPs with extreme discriminant scores (mean ± 2 standard deviations) were selected, leading to a final set of 75 SNPs.

To assess the effectiveness of the selected SNPs in separating the British Lop from the other breeds, cluster analyses were performed on each of the validation sets based on the genomic relationship matrix estimated from these 75 SNPs, using the GEMMA software [20]. Clusters were assessed both through visual inspection and using a Hierarchical Clustering method implemented in the R-package “FactoMineR” [42].

In addition, the predictive capacity of the final set of 75 SNPs was tested in a series of Canonical Discriminant Analyses, using the genotypes as predictive variables and the binary outcome (British Lop or other) as the response variable. These analyses were performed using the R-package “MASS” [41], with the model being fit to the training dataset and then used to predict the response variable for the animals in the validation datasets.

## Results

### Genomic characterization of the British Lop

Estimates of genetic diversity were reflective of a population with a relatively small effective size and under significant drift effects. The population showed a low nucleotide diversity average of 5.53e-06 per Mb (over 2,253 bins across the entire genome), with a standard deviation of 2.84e-06 and minimum and maximum values of 5.68e-09 and 1.57e-05, respectively. This nucleotide diversity was lower than the observed for Large White (6.37e-06 per Mb), Pietrain (6.23e-06 per Mb), Welsh (6.16e-06 per Mb) and Landrace (5.83e-06 per Mb), but slightly higher than for Middle White (5.47e-06 per Mb).

The estimate of Tajima’s D in British Lop was positive and significantly different from zero (beta distribution and 95% confidence limit for 175 samples, as in Tajima [43]), with an average of 2.56 for the entire genome and a standard deviation of 1.26. Nucleotide diversity and Tajima’s D showed no significant differences among chromosomes, potentially indicating minor effects of directional selection compared to genetic drift (S1 Fig).

**Fig 1:**
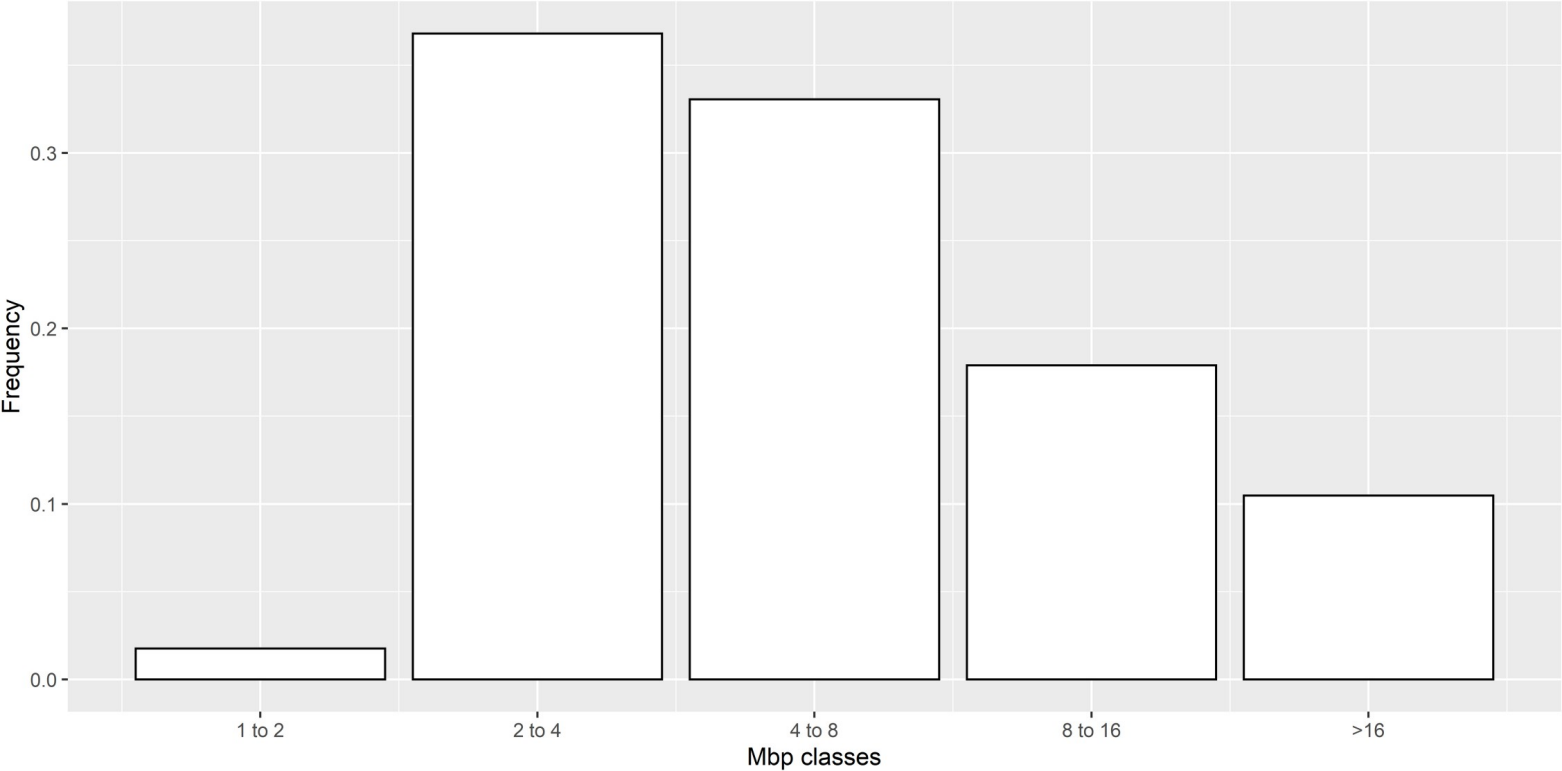
Frequencies of Runs of Homozygosity (ROH) according to segment size in Mb. ROH were calculated for 176 British Lop samples and 44,315 SNPs and classified based on their length.

The ROH analysis detected 11,306 ROH, with an average size of 8,073 Kb and a standard deviation of 9,713 Kb. Individual ROH varied largely in size (from 1,340 to 178,513 Kb). On average, 64.2 ROH were detected per individual with a standard deviation of 7.6 and minimum and maximum of 24 and 83, respectively. Fig 1 illustrates the ROH frequencies when classified by segment size into five classes corresponding to lengths of 1–2 Mb, 2–4 Mb, 4–8 Mb, 8–16 Mb and >16 Mb [10].

Inbreeding estimates in the British Lop population are summarized in Table 1. Although high positive correlations (>0.9) were observed among the different genomic estimates of inbreeding, correlations with pedigree-based estimates were relatively low (<0.4), possibly because the pedigree did not span the same time depth for all studied animals. On average, the available pedigree spanned 2.1 complete generations. Pedigree also spanned on average 4.7 maximum generations (the generations separating an individual from the most distant known ancestor). While the number of maximum generations ranged from zero to eleven across all animals (S2 Fig), nearly one third of the animals had pedigree data spanning fewer than four generations and about 8% had no pedigree information at all.

**Table 1:** Estimates of inbreeding for the genotyped British Lop animals.

Method	Average	Standard error	Minimum	Maximum
**Pedigree**	0.033	0.004	0	0.264
**VanRaden** ^**1**^	0.291	0.003	0.112	0.412
**Nejati-Javaremi** ^**2**^	0.645	0.002	0.556	0.706
**ROH** ^**3**^	0.230	0.004	0.067	0.384

^1^Based on allelic frequency change, assuming frequencies of 0.5 in the base population to correct for IBS [26].

^2^Proportion of homozygosity in the genome accounts for IBS and IBD [28].

^3^Based on runs of homozygosity.

**Fig 2:**
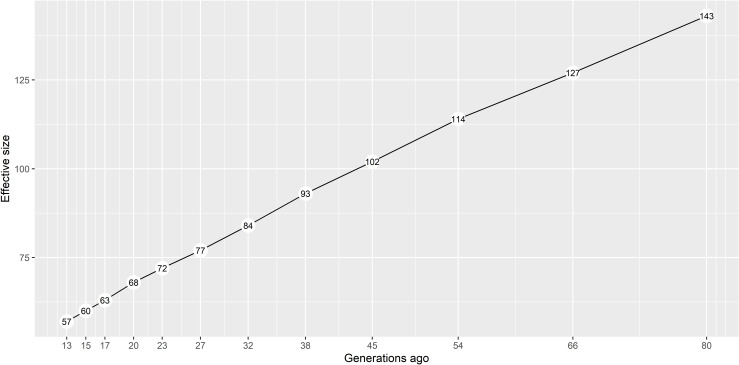
Historical effective size trend in the British Lop population. Calculated based on a dataset of 176 genotypes and 36,048 SNPs, using the SNeP software and the mapping function of Sved & Feldman.

The historic effective population size trend estimated with SNP information for the British Lop is presented in Fig 2. This trend shows a linear decrease with no sudden recent bottlenecks and is consistent with a smooth reduction in size expected from genetic drift in a numerically small and isolated population. As estimates for earlier generations may not be very reliable, a linear model was fitted (R^2^ = 0.999) to the most recent 54 generations (equivalent to an average bin length of around 1Mb), indicating that the current estimated effective size of the population would be approximately 40. This genomic estimate of the current effective population size was in line with a pedigree-based estimate of around 45, associated with an observed increase in inbreeding of animals with complete generations.

### Relatedness among breeds

**Fig 3:**
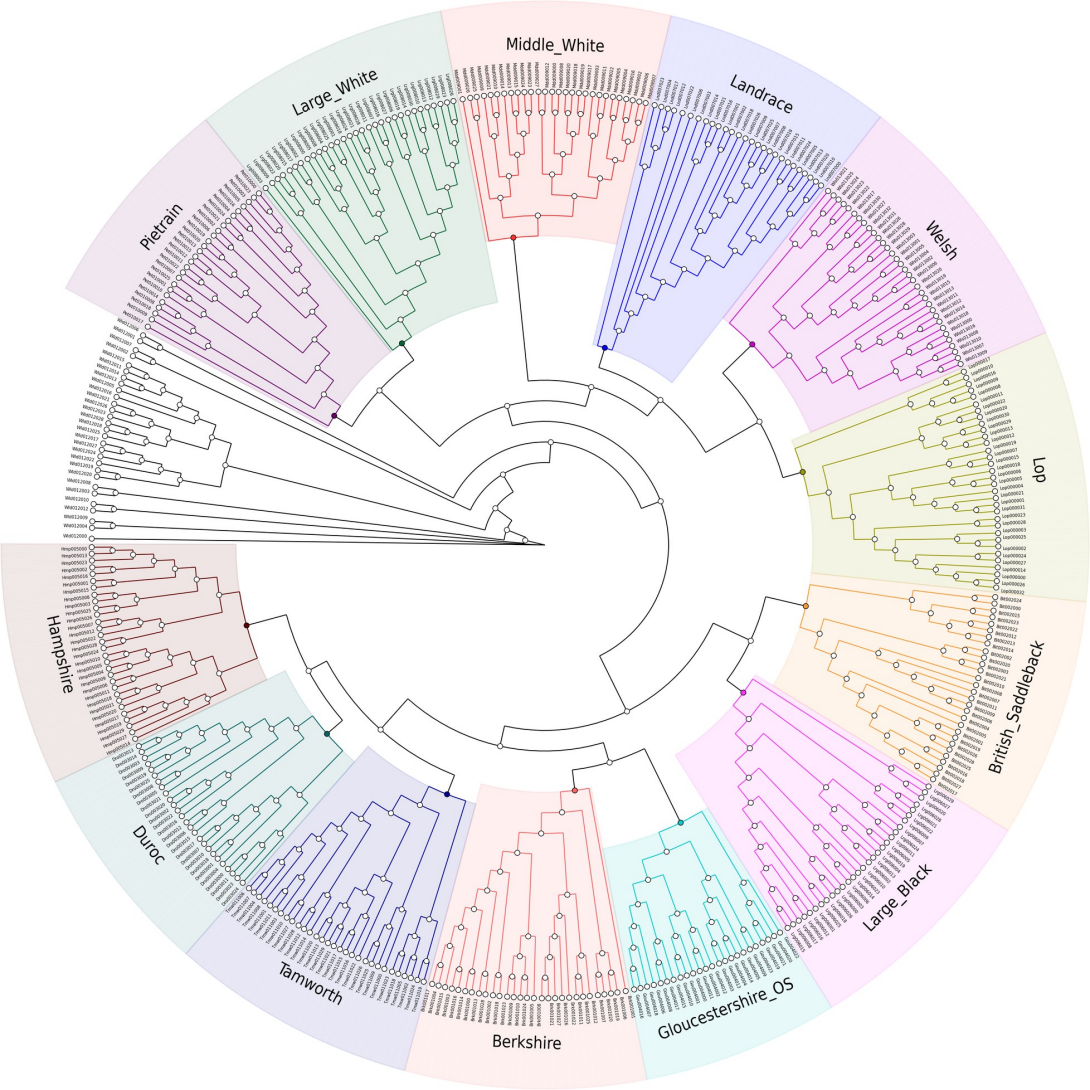
Neighbor-joining Identity-by-State (IBS) consensus tree for 14 pig populations. IBS genetic distances for 405 samples and 14,628 SNPs were estimated in PLINK with 100 bootstrap permutations, using PHYLIP and GraPhlAn to create the tree. Wild boar (in black) was used as outgroup.

The consensus tree based on IBS distances is presented in Fig 3. All animals used in this analysis were found to be correctly assigned to their respective breeds. All nodes at or above breed level had bootstrap values over 95%, except for three cases: the node separating Middle White from the Landrace, British Lop and Welsh cluster (65%), the node separating the Tamworth, Duroc and Hampshire cluster from the Berkshire and Gloucester Old Spots cluster (69%), and the node separating the British Saddleback and Large Black cluster from the Tamworth, Duroc, Hampshire, Berkshire and Gloucester Old Spots cluster (73%). The closest breeds to the British Lop according to this analysis were Landrace and Welsh, followed by Middle White, Large White and Pietrain.

**Fig 4:**
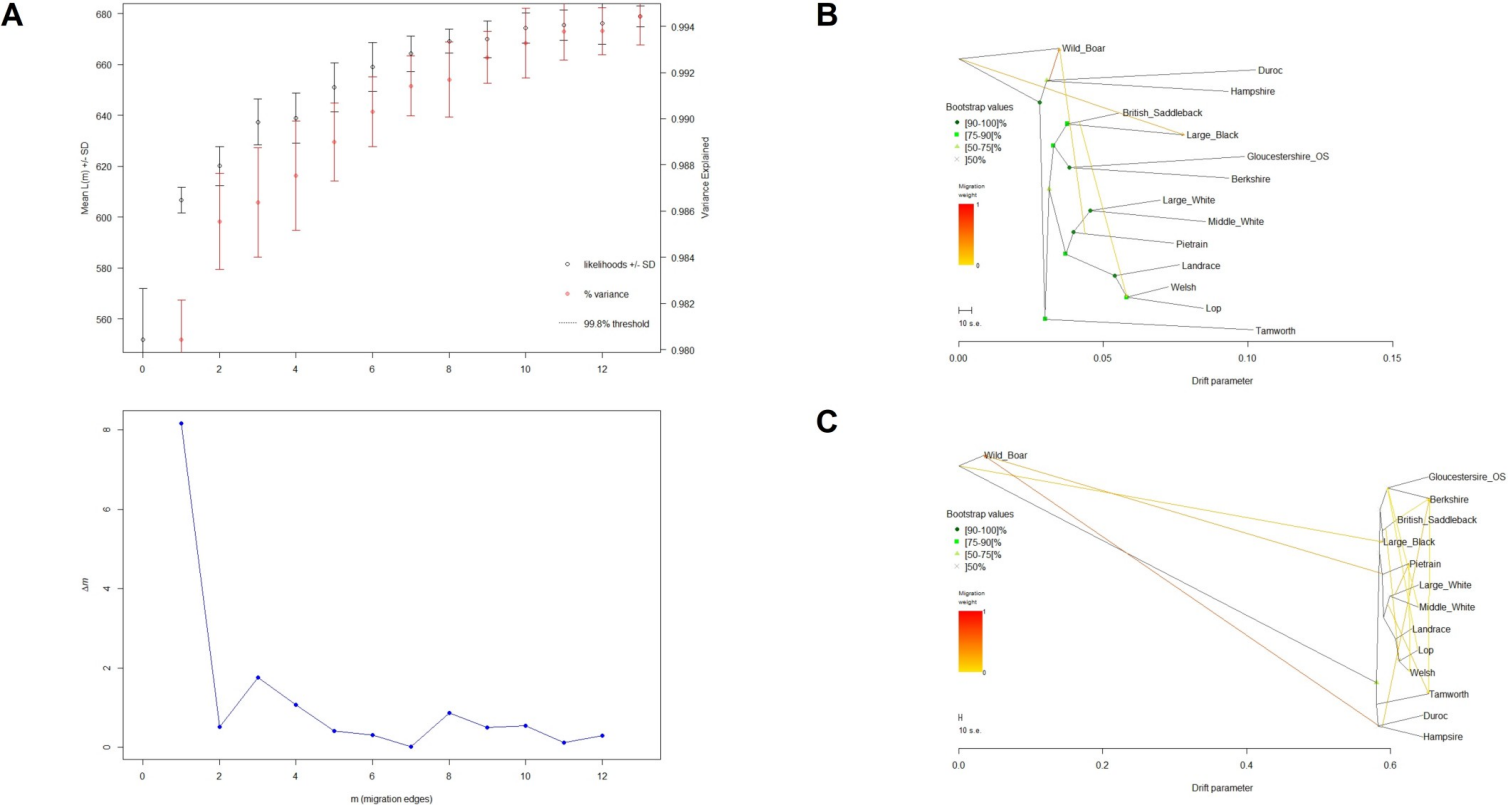
Evanno analyses (A) and maximum-likelihood consensus trees with 4 (B) and 12 (C) migration edges for 14 pig populations. For a total of 405 samples and 14,628 SNPs, allelic frequencies per breed were estimated in PLINK, using Treemix to create the trees. The Evanno method (10 bootstrap replicates per node number) was used to estimate the optimal number of migration nodes; 12 migration edges explained 99.4% of the variance, while 4 migration edges explained 98.6% of the variance.

Preliminary Evanno analyses aiming to identify the optimal number of potential migration patterns between breeds showed that 12 edges in the maximum-likelihood tree would explain 99.4% of the variance (Fig 4A). However, considering only 4 migration edges instead would still explain 98.6% of the variance and, at the same time, allow for a more meaningful interpretation of the tree compared to the complexity associated with 12 edges. Therefore, consensus trees with both 4 and 12 migration nodes were considered to examine possible migration patterns (Fig 4B and 4C, respectively). None of these trees showed significant migrations affecting the British Lop, and both corroborated the IBS results, thereby confirming that the closest breeds to the British Lop were Landrace and Welsh, followed by Middle White, Large White and Pietrain.

**Fig 5:**
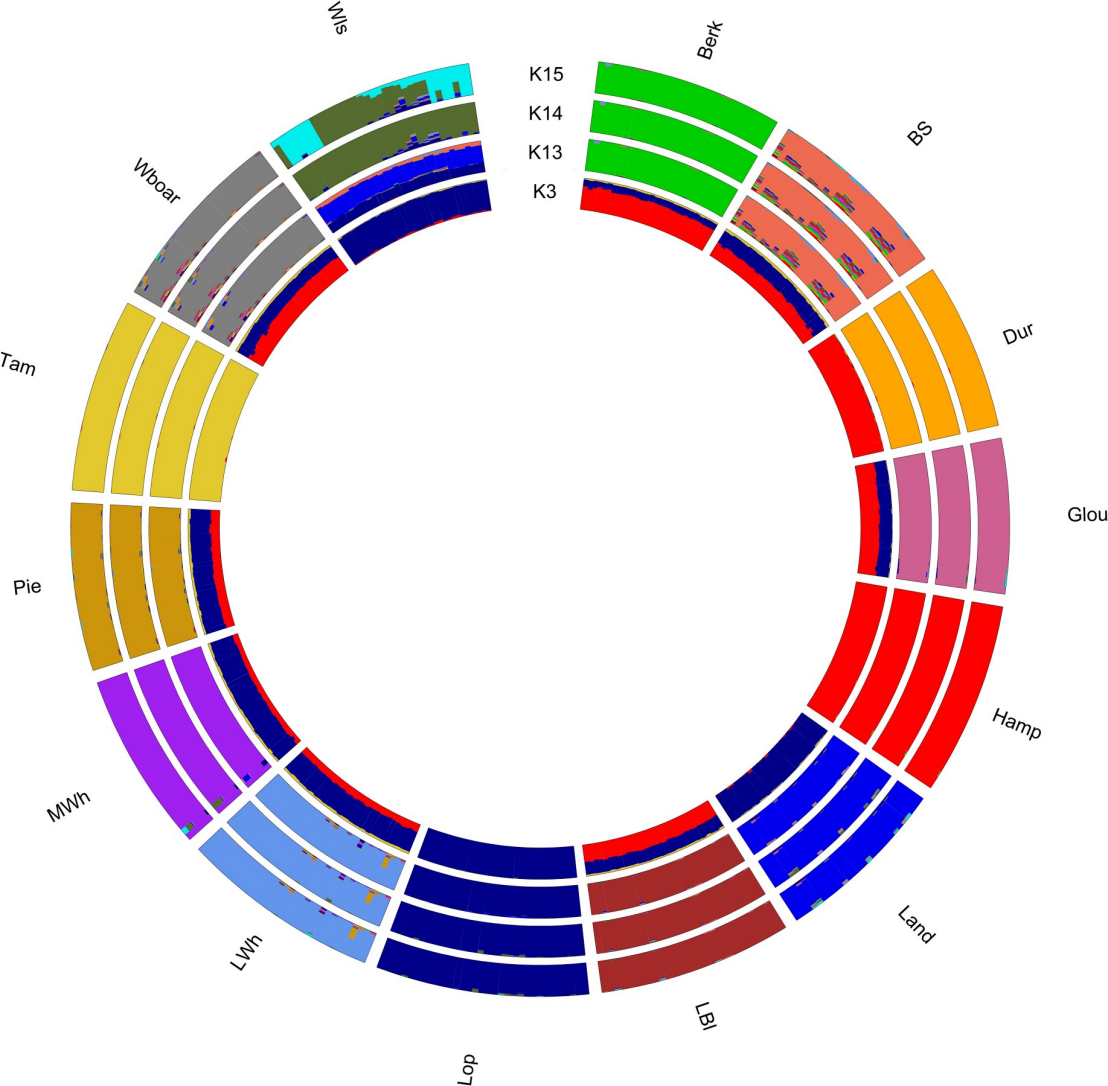
Unsupervised admixture analysis of the studied pig populations for 3, 13, 14 and 15 ancestral populations (K3, K13, K14, K15). For a total of 405 samples and 14,628 SNPs. Admixture levels were estimated using the ADMIXTURE software. Error estimates were calculated using a 5-fold cross-validation to estimate the most likely value for the number of ancestral populations (K). The label was given a posteriori by comparing the membership coefficient with the known breed. As such, cluster codes are: Berkshire (Berk), British Saddleback (BS), Duroc (Dur), Gloucestershire Old Spots (Glou), Hampshire (Hamp), Landrace (Land), Large Black (LBl), Large White (LWh), Lop (Lop), Middle White (MWh), Pietrain (Pie), Tamworth (Tam), Welsh (Wls) and Wild boar (Wboar).

The unsupervised admixture analysis (Fig 5) resulted in an optimum number of 15 ancestral populations based on the smallest cross-validation error (0.49661 after 20 iterations). The 15^th^ population was the result of an underlying substructure in the Welsh samples. Relevant admixture proportions are shown in Table 2. Very low admixture (1.68%) was observed in the British Lop population, primarily emanating from the Welsh (1.20%) and Landrace (0.42%). Admixture levels in the other breeds of study ranged from 0.09% in Duroc to 22.51% in British Saddleback. Interestingly, the cross-validation error for 14, compared to 15, ancestral populations was only marginally greater (0.49778 after 16 iterations), potentially indicating that the observed substructure in the Welsh sample was very light. Fig 5 also shows the results of considering 13 and 3 ancestral populations. The analysis of 13 ancestral populations (cross-validation error of 0.50448 after 17 generations) included all populations except Welsh. Assuming only 3 ancestral populations, a clear genetic distinction of the European lop-eared breeds (British Lop, Welsh and Landrace) can be seen from the Duroc and Hampshire cluster and from Tamworth.

**Table 2:** Average admixture in the British Lop and other pig populations (unsupervised admixture analysis).

Breed of study	Average proportions from ancestral breeds (%; rounded to 2 decimal places)														
	British Lop	Berk	BS	Dur	Glou	Hamp	Land	LBl	LWh	MWh	Pie	Tam	Wboar	WlsA	WlsB
**British Lop**	98.32	0.00	0.01	0.00	0.03	0.00	0.42	0.01	0.00	0.00	0.00	0.00	0.00	1.10	0.10
**Berk**	0.12	99.05	0.15	0.06	0.14	0.01	0.05	0.02	0.31	0.03	0.00	0.05	0.00	0.00	0.00
**BS**	0.37	3.41	77.49	0.36	1.85	1.66	1.73	4.84	1.81	0.93	1.65	0.92	2.12	0.30	0.55
**Dur**	0.00	0.00	0.00	99.91	0.00	0.08	0.00	0.00	0.00	0.00	0.00	0.00	0.00	0.00	0.00
**Glou**	0.12	0.00	0.04	0.00	99.13	0.00	0.00	0.00	0.14	0.07	0.00	0.00	0.00	0.12	0.38
**Hamp**	0.00	0.04	0.00	0.04	0.02	99.88	0.00	0.00	0.00	0.00	0.00	0.00	0.00	0.00	0.00
**Land**	1.24	0.12	0.49	0.00	0.24	0.02	94.90	0.00	0.68	0.31	0.25	0.06	0.10	0.54	1.05
**LBl**	0.06	0.08	0.36	0.00	0.29	0.14	0.18	98.46	0.06	0.05	0.04	0.05	0.03	0.04	0.17
**LWh**	0.29	0.20	0.07	0.28	0.31	0.07	0.40	0.16	95.19	0.52	1.70	0.16	0.16	0.25	0.23
**MWh**	0.22	0.02	0.00	0.00	0.12	0.00	0.10	0.00	0.07	98.15	0.00	0.02	0.00	0.70	0.59
**Pie**	0.24	0.17	0.25	0.09	0.12	0.05	0.27	0.08	0.22	0.01	97.73	0.21	0.05	0.14	0.38
**Tam**	0.00	0.02	0.05	0.10	0.01	0.04	0.03	0.02	0.00	0.00	0.00	99.69	0.00	0.00	0.04
**Wboar**	0.61	0.30	0.57	0.50	0.65	0.43	0.55	1.23	0.90	0.59	0.93	0.55	91.20	0.69	0.31
**Wls**	2.71	0.07	0.13	0.02	0.18	0.05	2.14	0.14	0.49	0.13	0.28	0.15	0.17	50.23	43.12

Berkshire (Berk), British Saddleback (BS), Duroc (Dur), Gloucestershire Old Spots (Glou), Hampshire (Hamp), Landrace (Land), Large Black (LBl), Large White (LWh), Middle White (MWh), Pietrain (Pie), Tamworth (Tam), Wild boar (Wboar) and Welsh (Wls). WlsA and WlsB are detected subpopulations of Welsh.

### Identification of a unique SNP set distinguishing the British Lop

The third aim of this study was to identify SNPs that would consistently distinguish British Lops from the closest related UK breeds identified in the previous analyses (Landrace, Welsh, Middle White, Large White and Pietrain).

The starting set of 3,417 LD pruned variants was reduced to 630 SNPs based on pairwise F_st_ comparisons. Canonical Discriminant Analyses were then performed within each chromosome, further reducing the set of unique SNPs to 75.

In all subsequent validation analyses, the use of Hierarchical Clustering methods with these 75 SNPs consistently separated the British Lop cluster from other breed clusters, with the two known British Lop outliers correctly placed outside the British Lop cluster. Furthermore, the Canonical Discriminant Analyses conducted to test the predictive ability of these SNPs always identified correctly an animal as British Lop or not.

The names and positions of the selected 75 SNPs are provided in the S1 Table. Aggregate genotypes per chromosome for these 75 SNPs are presented in Table 3, including the most common allelic combination in British Lop and its frequency in the other populations. Some of these aggregate genotypes, particularly on chromosomes 1 to 4, had very low frequency in the other populations. Furthermore, aggregate genotypes observed in the British Lop were, almost never, the most frequent ones in the other populations. The SNPs in Table 3 should be collectively considered to distinguish a British Lop animal and could be used to develop a breed purity test.

**Table 3:** Aggregate genotypes for the selected 75 SNPs per autosomal chromosome that, combined, distinguish the British Lop.

Chromosome	Block size (number of SNPs)	Most frequent aggregate genotypes per chromosome in British Lop	Frequency in British Lop	Frequency in the other breeds
1	7	GCGCCGT	0.304	<0.001
2	8	TCACTAAC	0.241	<0.001
3	13	TTATTGGGACCGT	0.298	<0.001
4	10	ACCGTTGAGA	0.261	<0.001
5	4	TTCA	0.701	0.144
6	1	-	-	-
7	3	ATA	0.744	0.089
8	3	GTG	0.304	0.138
9	4	GGTC	0.518	0.036
10	1	-	-	-
11	3	ACG	0.664	0.195
12	2	CC	0.803	0.221
13	4	GCAA	0.412	0.04
14	3	TAG	0.767	0.102
16	3	TTC	0.768	0.227
17	4	GGTA	0.757	0.011
18	2	AC	0.434	0.057

Allele coding is according to the Illumina Forward strand calling system from the 10.2 genome assembly.

## Discussion

The development of worldwide intensive livestock production systems has stimulated rigorous genetic selection and the formation of a few high producing commercial breeds to respond to increasing market demands. This, in turn, has led to the decline of many native pig breeds in recent years. However, the conservation of rare pig breeds is extremely important as they constitute useful reservoirs of genetic variability. Such breeds may also possess certain desirable traits for niche markets and/or be of cultural heritage importance.

The focus of the present study was on the British Lop, which serves as a typical example of a rare pig breed. The genomic characterization of this breed revealed a small effective population size and possible strong effects of genetic drift. The nucleotide diversity was lower than in other breeds and Tajima’s D values were positive and significantly different from zero. While previous studies on mitochondrial DNA [13] have reported negative values of Tajima’s D in populations under genetic drift conditions, differences between mitochondrial and nucleic DNA diversity patterns have documented in breeds with small effective population size [44].

Genomic inbreeding and runs of homozygosity in the British Lop were in line with other rare autochthonous European breeds such as Apulo-Calabrese, Casertana and Nero Siciliano [9, 10]. The estimated average genomic inbreeding was larger than those reported for commercial breeds such as Landrace and Yorkshire [25], which is probably reflective of the small effective population size of the British Lop. The average homozygosity in British Lop of 64%, derived from the coefficients of the genomic relationship matrix under the allelic similarity method [28], was similar to reported estimates on other European native breeds [9] and highly correlated with the genomic inbreeding coefficients reported here based on the VanRaden methods [26, 27].

Admittedly, the commercial array used in the present study for animal genotyping may have underrepresented rare variants pertaining to rare breeds. This could affect the estimation of genomic homozygosity levels [45]. Nevertheless, consistency of our results with outcomes from previous studies on other rare pig breeds based on different commercial arrays [9] lends credibility and suggests a limited impact of ascertainment bias.

Pedigree-based inbreeding reported in the present study was probably an underestimate due to lack of complete historic pedigree data. Therefore, our estimates of genomic inbreeding and homozygosity are collectively of higher value for the management of this genetic resource than the estimates based on pedigree.

Estimates of the effective population size in the British Lop were generally similar to those reported in other rare breeds in previous studies [9]. However, our estimates (40–45) were lower than those reported by the UK Farm Animal Genetic Resources Inventory 2020 [14] for the British Lop. The latter reported an effective population size of 94 calculated as *N*_*e*_ = (4*N*_*sires*_ *N*_*dams*_) / (*N*_*sires*_ + *N*_*dams*_), relying on the assumptions of random mating, non-overlapping generations and a Poisson distribution of number of offspring [46]. In the studied population, however, these assumptions do not apply and the above equation would overestimate the effective population size. Thus, estimates reported in the present study should more accurately reflect the population structure and mating practices of the British Lop.

The above results of the genomic characterization of the British Lop are in line with the current status of a priority breed at risk [4]. With only about 161 sows being registered in the UK in 2019 [13], the small effective population size reported here implies a relatively strong risk of endangerment according to the 50/500 rule proposed by Franklin [47]. Although this rule has been contested [48], recent studies suggest that purging of lethal and non-lethal alleles must be considered [49, 50] in genetic management programs of numerically small populations, as discussed later.

Landrace and Welsh were the two breeds most closely related to the British Lop. These results are concordant with both the origin of the breed from lop-eared white breeds, like the Welsh, in the southwest of the UK [13] and with previous studies indicating possible introgression from British Landrace lines [51]. Furthermore, a cluster of other white breeds (Large White, Middle White and Pietrain) previously documented [52] was also relatively closely related to British Lop. However, despite the similarities with Welsh and Landrace and potential introgression from other breeds [51, 53], no significant migrations into the British Lop were identified and very little admixture was detected (about 1% admixture with Welsh and less than 0.5% with Landrace). These results support the notion that the British Lop has been maintained in relative isolation from other breeds.

As in previous studies [52], Hampshire and Duroc cluster together and separately from the British Lop and other breeds, probably because they are North-American breeds derived from European pigs. Furthermore, certain breeds such as Large Black, Gloucester Old Spots, Berkshire, British Saddleback and Tamworth were clearly separated from the British Lop, in line with previous studies [52]. This distinction is potentially linked to historic crossbreeding of the former breeds with Neapolitan and Asian pigs [16]. To this effect, we performed an additional admixture analysis (data not shown) including publicly available autosomal genotypes from two Asian breeds (24 Meishan and 11 Jiangquhai). This analysis revealed no admixture of the British Lop with the Asian breeds. Only the British Saddleback and Large White showed low levels of admixture (1%) with these two breeds. Further investigation of possible admixture of other UK and European rare breeds with Asian populations would certainly be of interest but beyond the scope of the present study.

A small number of SNPs were identified in the present study that distinguish British Lop pigs from other UK breeds. These SNPs may enable traceability of British Lop animals and their products. The development and practical implementation of this genomic tool can be useful for farmers interested in testing animals with unknown or uncertain origin. Based on our results, relevant prediction accuracy is expected to be 100%, thereby attesting to their utility in developing a breed purity test. In such a case, possible changes in allelic frequencies in future generations need to be considered. While in the present study the selected SNPs exhibited high variability, genetic drift leading to random changes in the allelic frequencies may be expected, thereby affecting their efficacy in future generations. Therefore, a re-evaluation of the set of distinctive markers is recommended every few generations. This is common practice in genomic selection and management programs. In this respect, the methodology described here is relatively simple and can be easily implemented at a low cost.

As previously discussed, the present study used a commercial SNP array for animal genotyping that may have underrepresented certain rare variants in the British Lop and other rare breeds. Nevertheless, our results suggest that this array is still suitable for the identification of the unique SNPs distinguishing the population of study. Various commercial arrays have also been used successfully in previous genomic studies of other rare breeds of pigs [5, 12, 52] and other species [54, 55].

Results of the present study may collectively inform genetic management strategies for the British Lop considering genetic drift and inbreeding depression, while maintaining the unique characteristics of the breed and potential rare variants of interest. Regarding the latter, whole-genome sequencing would be a useful, albeit costly, approach. A potential alternative would be to use the existing genome-wide SNP arrays to identify rare segregating haplotypes in the breed. Admittedly, in a population under strong genetic drift these regions could be associated to deleterious mutations segregating in the genome. Therefore, rare haplotypes should be considered in association with fitness-related animal traits.

Genetic management strategies of the breed would vary depending on the objective of the breeding program. Under a conservation approach, strategies such as equal contributions of parents can be applied to minimize global co-ancestry and assure all breeding animals contribute to the ensuing generations [56, 57]. Some studies proposed the use of inbred matings to purge deleterious segregating alleles [58, 59]. Although this is not recommended in the short term, as the potential benefit may not counteract the increased inbreeding depression on fitness traits [49, 60], relaxation of the practices in the long term could allow purging of undesirable alleles segregating naturally [61]. If the objective of the breeding program includes genetic improvement of animal traits of interest, more options may be available. For example, after an initial period of equal contributions to increase genetic diversity, optimal contribution strategies could be implemented to simultaneously minimize inbreeding and achieve genetic gain in the desired traits [62]. Further relevant strategies in this regard may consider alternative markers such as Copy Number Variations (CNV) that have been associated with positive effects on reproductive traits in Asian breeds [63] and body conformation traits in other breeds [64]. However, at the moment CNV-based solutions report high false positive rates [65] and are viewed as less practical than SNPs for day-to-day management practices.

In conclusion, the present study of the British Lop as a typical example of a numerically small autochthonous pig breed, revealed relatively high levels of inbreeding and reduced genetic diversity compared to larger commercial breeds. Despite previous reports of potential introgression from other breeds, very little admixture has been detected, even with closely related breeds such as Landrace and Welsh. A set of 75 SNPs was identified that, combined, uniquely characterize the British Lop and may be used to develop a breed purity test to distinguish them from other breeds with similar phenotypic characteristics. This low-cost genomic tool may be used in conjunction with other management practices for the future conservation, maintenance and improvement of the breed. Moreover, such a tool could also be commercially utilized by food inspection agencies for product traceability and consumer protection.

## Supporting information

S1 Fig Nucleotide diversity (A) and Tajima’s D (B) estimates in the British Lop population per chromosome. Boxplots per chromosome for nucleotide diversity and Tajima’s D, estimated in windows of 1 Megabase using 176 British Lop samples and 44,315 SNPs. For nucleotide diversity, genome-wide minimum and maximum values were on chromosomes 1 and 8 (5.68e-09 and 1.57e-05, respectively), and chromosomal average ranged from 4.393e-06 (chromosome 15) to 6.773e-06 (chromosome 17). For Tajima’s D, genome-wide minimum and maximum values were detected on chromosomes 5 and 14 (-2.015 and 5.554, respectively), and chromosomal average ranged from 2.132 (chromosome 11) to 2.916 (chromosome 17).
(TIF)

S2 Fig Frequencies of maximum generations in the British Lop population. The maximum number of generations traced for each individual was calculated based on the existing pedigree of 2,901 animal records including historic data.
(TIF)

S1 Table Selected 75 SNPs that, combined, distinguish the British Lop from other breeds. SNPs are presented per chromosome, indicating their position in base pairs and all the available calling systems (Illumina Forward, Illumina Top and Illumina AB). Forward strand allele calls correspond to the genome assembly 10.2; map positions have been updated accordingly with the *Sus scrofa* 11.1 assembly.
(XLSX)
