# Supplementary figures and images for: Genomic analysis of the rare British Lop pig and identification of distinctive genomic markers
Source: PLoS One. 2022 Aug 12;17(8):e0271053. doi: 10.1371/journal.pone.0271053 (PMC9374264; doi:10.1371/journal.pone.0271053)

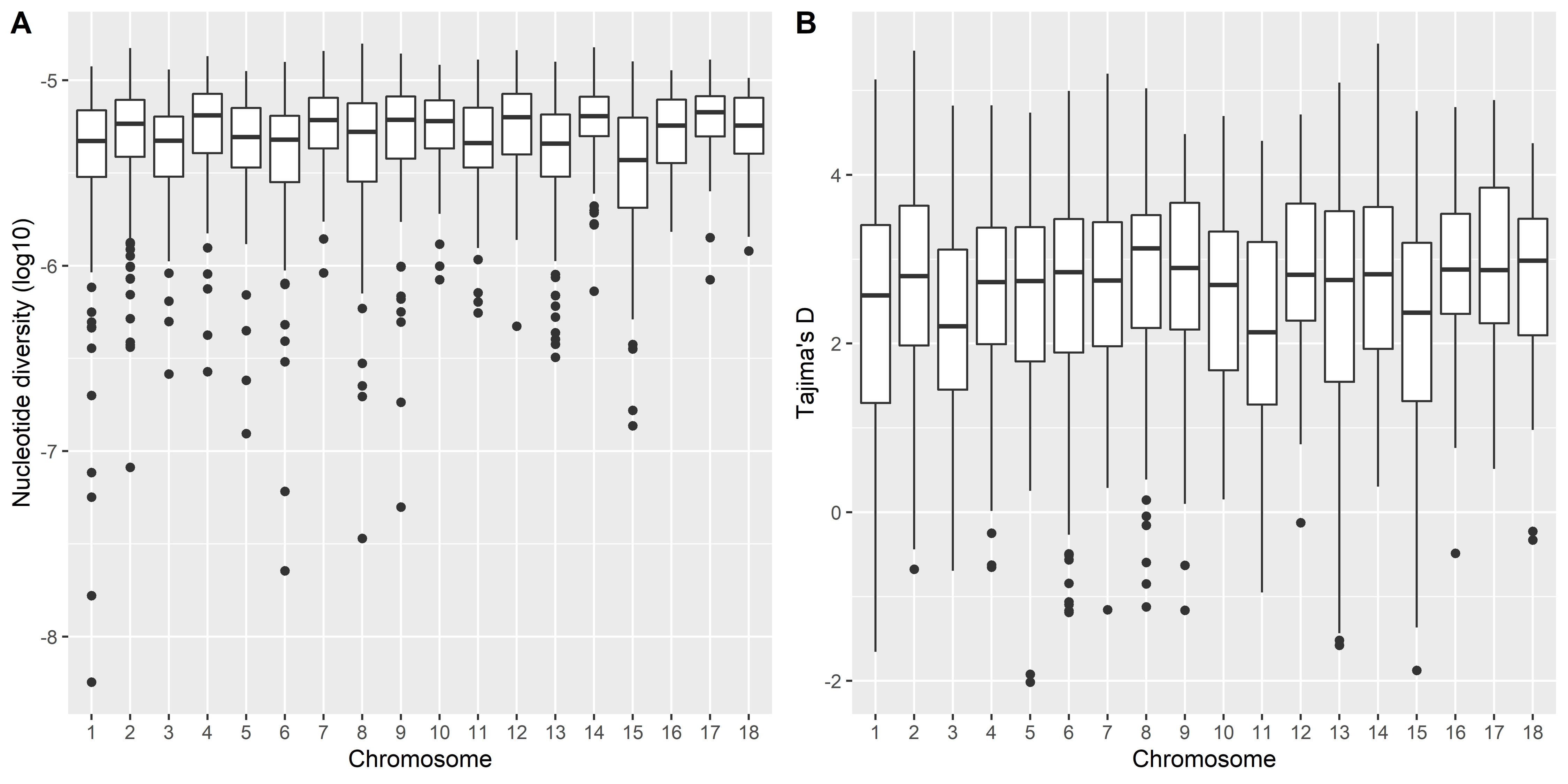

Supplement: S1 Fig — Nucleotide diversity (A) and Tajima’s D (B) estimates in the British Lop population per chromosome. Boxplots per chromosome for nucleotide diversity and Tajima’s D, estimated in windows of 1 Megabase using 176 British Lop samples and 44,315 SNPs. For nucleotide diversity, genome-wide minimum and maximum values were on chromosomes 1 and 8 (5.68e-09 and 1.57e-05, respectively), and chromosomal average ranged from 4.393e-06 (chromosome 15) to 6.773e-06 (chromosome 17). For Tajima’s D, genome-wide minimum and maximum values were detected on chromosomes 5 and 14 (-2.015 and 5.554, respectively), and chromosomal average ranged from 2.132 (chromosome 11) to 2.916 (chromosome 17). (TIF) [file pone.0271053.s001.tif]

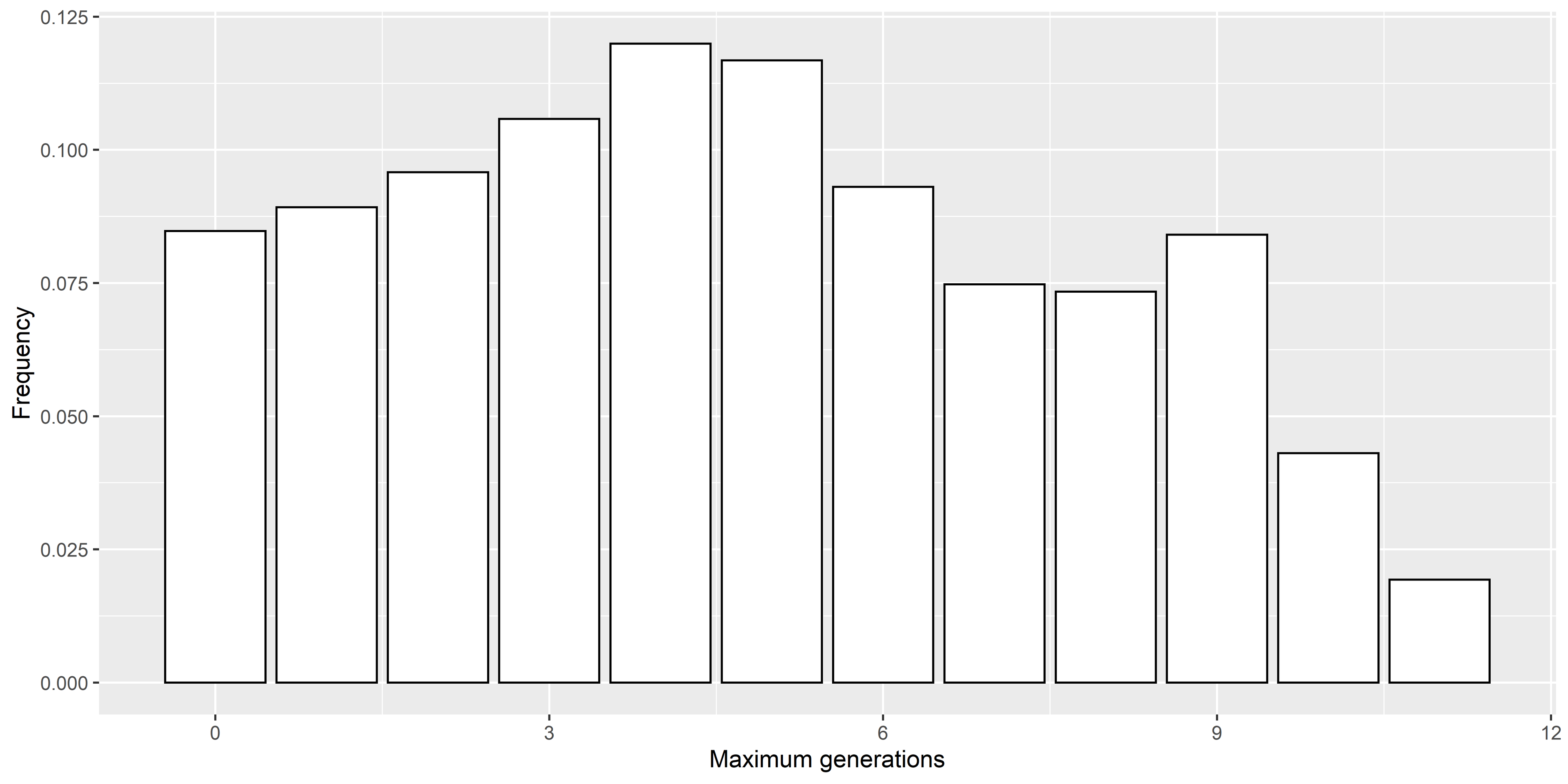

Supplement: S2 Fig — The maximum number of generations traced for each individual was calculated based on the existing pedigree of 2,901 animal records including historic data. (TIF) [file pone.0271053.s002.tif]
